# MG132 Ameliorates Kidney Lesions by Inhibiting the Degradation of Smad7 in Streptozotocin-Induced Diabetic Nephropathy

**DOI:** 10.1155/2014/918396

**Published:** 2014-01-05

**Authors:** Chenlin Gao, Keri Aqie, Jianhua Zhu, Guo Chen, Ling Xu, Lan Jiang, Yong Xu

**Affiliations:** ^1^Department of Endocrinology, Affiliated Hospital of Luzhou Medical College, Luzhou, Sichuan 646000, China; ^2^Department of Endocrinology, First People's Hospital of Liangshan, Xichang, Sichuan 615000, China

## Abstract

*Background*. Smad7 is the main negative regulatory protein in the transforming growth factor-**β** (TGF-**β**) downstream signaling pathway, which plays an important role in diabetic nephropathy (DN) and may be related to the ubiquitin proteasome pathway (UPP). *Aim*. We investigated the role of UPP in regulating TGF-**β**/SMAD signaling and explored the therapeutic effect of the ubiquitin proteasome inhibitor MG132 on DN. *Methods*. Wistar rats were randomly divided into a diabetes group and a normal control group. Rats in the diabetes group were injected intraperitoneally with streptozotocin. Diabetic rats were then randomly divided into a diabetic nephropathy group (DN group), an MG132 high concentration (MH) group, and an MG132 low concentration (ML) group. After 8 weeks of treatment, 24-hour urinary microalbumin (UAlb), urinary protein/urinary creatinine (Up/Ucr) values, ALT, AST, Bcr, kidney damage, TGF-**β**, Smad7, fibronectin (FN), and Smurf2 were detected. *Results*. The body mass and Smad7 protein expression decreased in DN group, but kidney weight, kidney weight index, UAlb, Up/Ucr, FN and Smurf2 mRNA expression, and TGF-**β** protein expression increased. However, these changes diminished following treatment with MG132, and a more pronounced effect was evident in MH group compared to ML group. *Conclusion*. MG132 alleviates kidney damage by inhibiting Smad7 ubiquitin degradation and TGF-**β** activation in DN.

## 1. Introduction

Diabetic nephropathy (DN) is one of the most prevalent and serious microvascular complications of diabetes mellitus (DM) [1]. Early pathological characteristics are basement membrane thickening, increase in mesangial matrix, and extracellular matrix accumulation, followed by development of glomerulosclerosis and tubulointerstitial fibrosis, eventually leading to irreversible renal damage [2–5]. The exact pathogenesis of diabetic nephropathy has not yet been completely clarified.

Smad7 is the main negative regulatory protein and antifibrotic factor in the transforming growth factor *β* (TGF-*β*) downstream signaling pathway [6] and can compete with Smad2/3 for binding to the type I TGF-*β* receptor, blocking Smad2/3 activation. Smad7 can also be transferred to the cell membrane for degradation of Smad2/3 and TGF-*β* receptor complexes, as well as inhibition of TGF-*β* signal activation after binding to the Smad ubiquitin regulatory factor 2 (Smurf2). Activation of TGF-*β* plays an important role in the pathological progress of diabetic nephropathy [7], which involves increased expression of many cytokines, inflammatory cytokines and adhesion molecules, induction of fibronectin (FN) expression [8, 9], and involvement in actual development of diabetic nephropathy [10, 11].

The ubiquitin proteasome pathway (UPP) is the main mechanism for intracellular protein degradation, and can degrade specific proteins, and regulate cell differentiation and transcription; Smurf is a ubiquitin ligase, which belongs to the E3 ligase family, and can specifically degrade Smad proteins. It has been determined that Smad proteins are degraded by a ubiquitin mechanism [12]. The Smurf ligase family includes Smurf1 and Smurf2. The function of Smurf2 is carried out via binding with the TGF-*β* receptor complex through Smad7, leading to ubiquitin degradation of Smad7, which weakens the inhibitory effect of Smad7 on the TGF-*β* receptor [6].

However, whether the UPP is activated or involved in the development of diabetic nephropathy in kidneys remains unclear. Research has shown that MG132 has therapeutic effects on diabetic nephropathy [13–15], but the mechanism by which it acts is unclear. The possibility that MG132 is able to inhibit activation of the TGF-*β* signaling pathway through blocking ubiquitin degradation of Smad7 in diabetic nephropathy has not been studied. Therefore, additional research to understand the relationship between the UPP and the TGF-*β* signaling pathway and the mechanism of action of MG132 in diabetic nephropathy is necessary. In this study, we established a rat model of diabetic nephropathy by using STZ and selected MG132 as the specific ubiquitin proteasome inhibitor for blocking the TGF-*β*/SMAD signaling pathway, in order to explore the relationship between the UPP and the TGF-*β*/SMAD signaling pathway in diabetic nephropathy.

## 2. Materials and Methods

### 2.1. Animal Model

A total of 45 male Wistar rats weighing 200 g were obtained from the Biotechnology Corporation of Teng Xing, ChongQing (China). Rats were kept in a special room with a stable ambient temperature of 18°C–22°C and housed in wire cages with free access to a standard diet and tap water for 7 days before the experiment. Blood glucose levels of all rats were measured prior to the experiment.

Rats were divided into two groups, namely, a control group (NC group, *n* = 10) and an experimental group (*n* = 35); diabetic rats in the experimental group were rendered diabetic by intraperitoneal injection of streptozotocin (Sigma-Aldrich, USA) at a dose of 60 mg/kg. Streptozotocin was dissolved in 0.1 M citrate buffer at pH 4.5. Meanwhile the rats in the NC group received, by intraperitoneal injection, the same volume of citrate buffer. After 3 days following the STZ injection, fasting glycemic measurements were performed in blood samples from tail veins; rats with a blood glucose level of ⩾16.7 mmol/L were confirmed as “diabetic,” and 4 weeks later, diabetic rats presented with mild microalbuminuria (an early sign of DN) and were included in the study. Diabetic rats were then further divided into three groups: diabetic nephropathy group (diabetic control, *n* = 10). ML group (treated with 0.05 mg/kg MG132 every day (CALBIOCHEM, USA), *n* = 10), and MH group (treated with 0.1 mg/kg MG132 every day, *n* = 10), Meanwhile, the NC and diabetic nephropathy groups received intraperitoneal injections of the same volume of citrate buffer every day.

### 2.2. Sample Collection and Body Weight and Kidney Weight Determination

All rats were weighed and 24-hour urine was collected every day. After 8 weeks of injections, rats were sacrificed after anesthetizing with pentobarbital (50 mg/kg, 1% concentration). Blood biochemistry was analyzed from collection of heart blood. Both kidneys were weighed and cut along the coronal plane; the upper poles of the right kidneys were used for pathology analysis, and the remaining parts of the right kidneys were used for transmission electron microscope analysis. Left kidneys were dissected for the assessment of biochemical parameters. Renal tissues were preserved at −80°C until required for analysis.

### 2.3. Biochemical Measurements

Measurements of 24-hour urinary microalbumin (UAlb), urine creatinine concentration, urine protein/urine creatinine (Up/Ucr) concentration and ALT, AST, TP, ALB, BUN, Crea, and GLU of blood were measured by an automatic biochemistry analyzer.

### 2.4. Kidney Pathology

The upper poles of the right kidneys were rapidly removed, fixed in 10% formaldehyde, dehydrated by gradient ethanol, embedded in paraffin, and sectioned at 4 **μ**m thickness. Renal sections were stained with HE and Masson staining. All sections were evaluated using a light microscope.

### 2.5. Kidney Transmission Electron Microscope

Renal cortices were cut into 1 mm pieces and fixed in 2.5% glutaraldehyde for 2 hours at 4°C. After being washed three times with 0.01 M phosphate buffer, samples were post-fixed in 1% osmic acid for 3 hours at 4°C. Samples were then dehydrated by gradient acetone and embedded in propylene oxide. Ultrathin sections (60 nm) were cut, double-stained with uranyl acetate and lead citrate, and examined with a transmission electron microscope (Philips Tecnai 10, USA).

### 2.6. Western Blot

Renal cortices were homogenized in lysis buffer (Kaiji, Shanghai, China) on ice for 30 minutes. Western blotting was performed as previously described [13]. Immunoblot analysis was performed using TGF-*β* antibody (rabbit, 1 : 1000; Cell Signaling Technology (CST), USA), Smurf2 antibody (rabbit, 1 : 1000; Abcam, USA), actin antibody (rabbit, 1 : 1000; Abcam, USA), Smad7 antibody (rabbit, 1 : 500; Boster Biological Technology, China). Horseradish peroxidase-conjugated secondary antibodies (anti-rabbit) were obtained from the Beyotime Institute of Biotechnology, China. Proteins were detected using the enhanced chemiluminescence (ECL) system and ECL Hyperfilm (Millipore, USA).

### 2.7. Real-Time Fluorescent Quantitative PCR

Total RNA was extracted from renal cortical homogenate using an RNA extraction kit (Tiangen Biotech, Beijing, China). PCR was performed as previously described [16]. The primer sequences were as follows: FN, forward: 5′-CATACTCCTCCAGACCTACC-3′, reverse: 5′-TGGAGGTTAGTGGGAGCATC-3′, Smad7, forward: 5′- CTGCAACCCCCATCACCTTA -3′, reverse: 5′-GCAACGCCTCCATAGTC-3′, actin, forward: 5′-TGGCATTGTCATGGACTCTG-3′ reverse: 5′-CCAGAAGAAGTTGGGAATCTGA-3′.

### 2.8. Statistical Analysis

All experimental data were expressed as means ± S.D. $\left(\overset{-}{x}\pm s\right)$. For statistical evaluation of the data obtained in our study, one-way analysis of variance (one-way ANOVA) was used to compare more than two groups, followed by Fisher's least significant difference (LSD) test for multiple comparisons, by using the statistical package SPSS 13.0; a *P* value of <0.05 was considered as statistically significant.

## 3. Results

### 3.1. Change in Body Weight, Kidney Weight, and Kidney Weight Index

Body weight was lower in the experimental groups compared with the NC group (*P* < 0.05), but it significantly recovered in the MH and ML groups compared with the diabetic nephropathy group (*P* < 0.05). Conversely, kidney weight and kidney weight index were greater in the experimental groups compared with the NC group (*P* < 0.05) but decreased upon MG132 treatment (*P* < 0.05). Changes in the MH group were more significant than in the ML group (*P* < 0.05) (Table 1).

### 3.2. Volume of Urine (UV), Urinary Albumin Excretion Rate (UAER), and Urine Protein/Urine Creatinine (Up/Ucr) Ratio

The UV, UAER, and Up/Ucr ratios were increased in the experimental groups compared with the NC group, and were the highest in the diabetic nephropathy group (*P* < 0.05). However, values were less in the MH and ML groups compared with the diabetic nephropathy group (*P* < 0.05); the MH group had even lower values than the ML group (*P* < 0.05) (Table 2). Meanwhile, there was not significant change of ALT, AST and Bcr in each group (*P* > 0.05) (Table 3).

### 3.3. Renal Pathology

**Figure 1:**
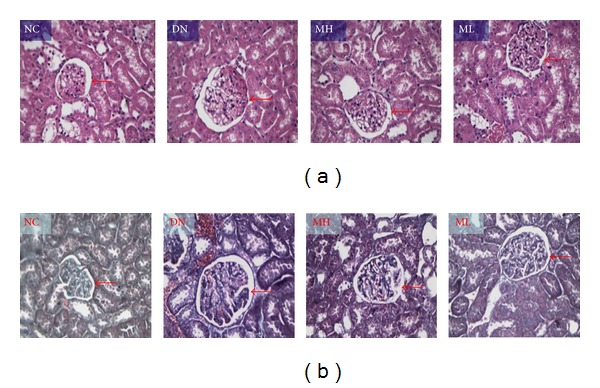
Changes of renal pathology observed by light microscopy. (a) HE staining (×200). (b) Masson staining (×200). The glomerular volume (red arrow) was greater in the experimental group than in the NC group, whilst renal tubular edema, abnormal glomerular mesangial deposition, and atrophy and degeneration of renal glomeruli were observed in the experimental groups, although changes were significantly less in the MH group.

The renal tissue volume in the experimental group is larger than in the NC group in gross appearance. After HE and Masson staining, we observed, by light microscopy, that the glomerular volume was greater in the experimental group compared to the NC group. In addition, renal tubular edema, abnormal glomerular mesangial deposition, and atrophy and degeneration of renal glomeruli were evident in the experimental groups, although changes were significantly less in the MH group (Figure 1).

### 3.4. Transmission Electron Microscopy of the Kidney Tissue

**Figure 2:**
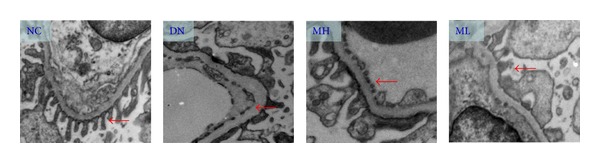
Transmission electron microscopy of the kidney tissue (×10,000). Hydropic endothelial cells and podocytes, endothelial pore broadening, irregular thickening and decrease in the electronic density of the basement membrane, and partial foot process fusion were visualized in the experimental groups (red arrow), especially in the diabetic nephropathy group, but were found to be recovered in the MH group.

Under the transmission electron microscope, we were able to visualize hydropic endothelial cells and podocytes, endothelial pore broadening, irregular thickening and decrease in the electronic density of the basement membrane, and partial foot process fusion in the experimental groups, especially in the diabetic nephropathy group, which were found to be recovered in the MH group (Figure 2).

### 3.5. Western Blot

**Figure 3:**
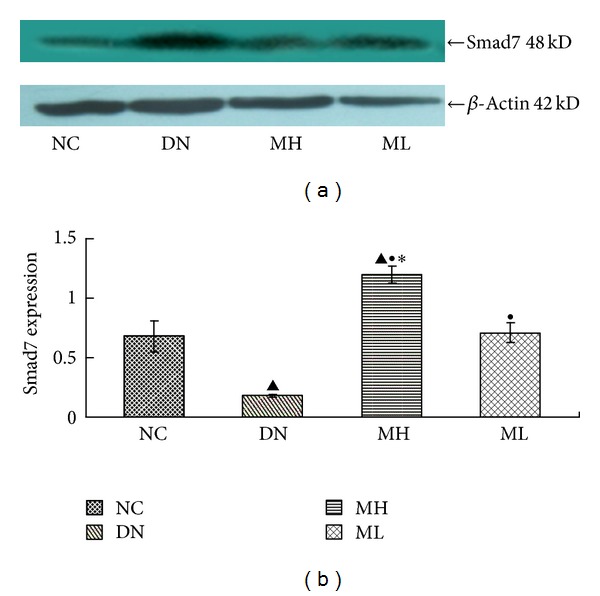
Smad7 protein expression in each group by Western blot. (a) Western blot strip chart. (b) The gray graph shows the relative statistical values of Smad7 for each group. Compared with the NC group, Smad7 expression decreased in the experimental groups, especially in the diabetic nephropathy group, with a subsequent increase in the MH and ML groups, and a more pronounced increase in the MH group compared with the ML group. ^▲^ *P* < 0.01 versus NC group, ^●^ *P* < 0.01 versus diabetic nephropathy group, and *P < 0.05 versus ML group.

**Figure 4:**
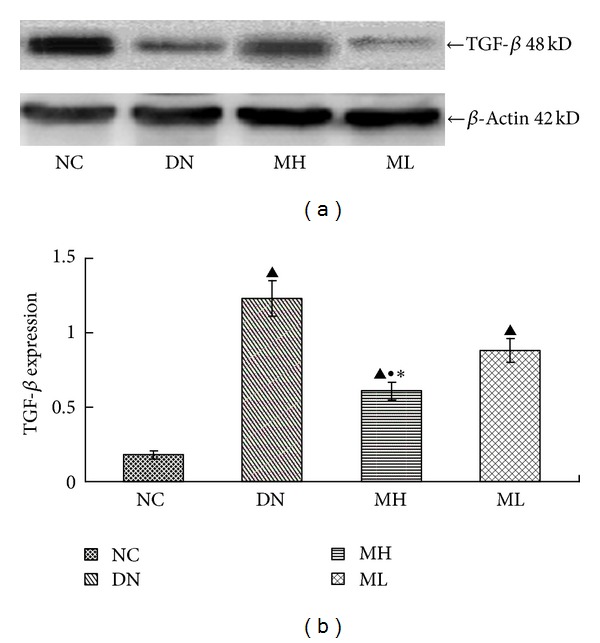
Expression of TGF-*β* protein in each group by Western blot. (a) Western blot strip chart. (b) The gray graph shows the relative statistical values of TGF-*β* for each group. Compared with the NC group, TGF-*β* expression increased in the experimental groups, especially in the diabetic nephropathy group, with a subsequent decrease upon MG132 treatment, and a more pronounced, significant decrease in the MH group.

There was a decreased expression of Smad7 in the experimental group compared with the NC group, particularly evident in the diabetic nephropathy group (*P* < 0.01), but Smad7 expression increased following MG132 treatment, and the increase in the MH group was more pronounced than in the ML group (*P* < 0.05). By contrast, TGF-*β* expression increased in the experimental group compared with the NC group, especially in the diabetic nephropathy group (*P* < 0.01), but MG132 treatment led to a decrease in TGF-*β* expression (*P* < 0.05), and there was a significantly lower expression of TGF-*β* in the MH group compared with the ML group (*P* < 0.05) (Figures 3 and 4).

### 3.6. Real-Time Fluorescent Quantitative PCR

**Figure 5:**
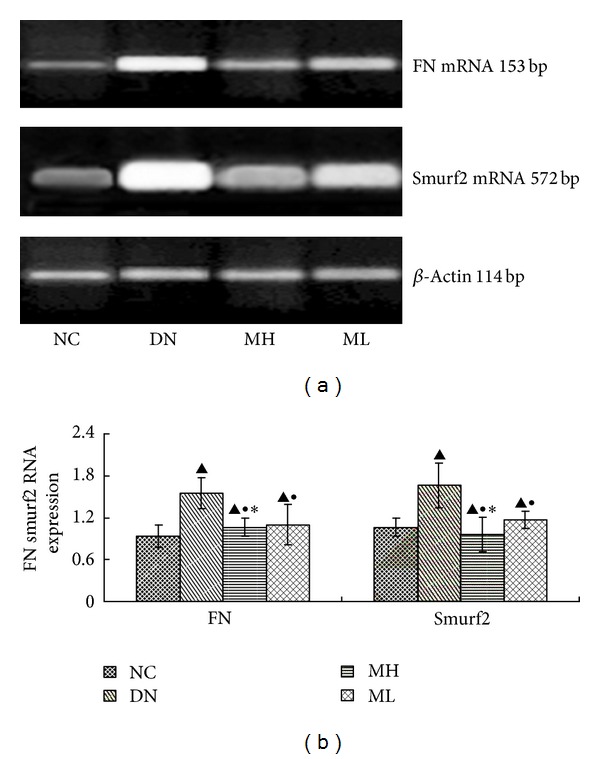
FN and Smurf2 mRNA levels by real-time fluorescent quantitative PCR in each group. (a) RT-PCR strip chart. (b) The gray graph shows the relative statistical values of the mRNA levels. Compared with the NC group, the levels of FN mRNA and Smurf2 mRNA increased in each experimental group, especially in the diabetic nephropathy group, but subsequently decreased in the MH and ML groups compared with the diabetic nephropathy group, with a more significant decrease in the MH group compared with the ML group.

**Figure 6:**
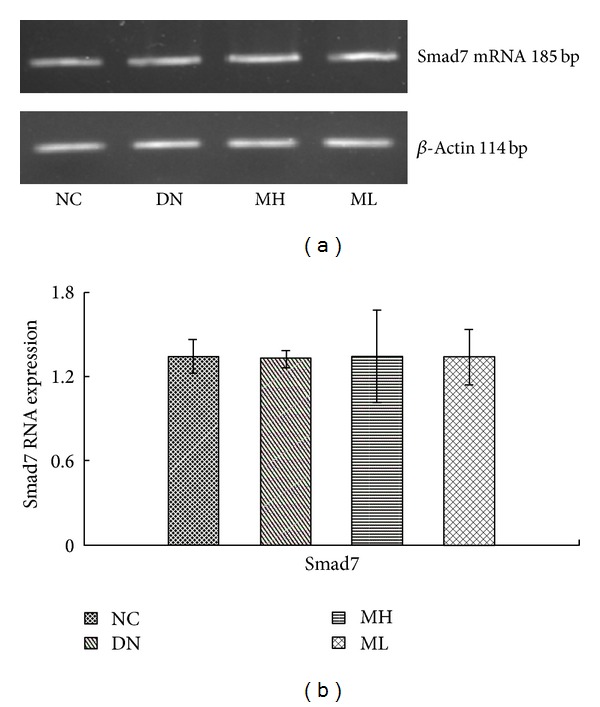
Smad7 mRNA levels by Real-Time fluorescent quantitative PCR in each group. (a) RT-PCR strip chart. (b) The gray graph shows the relative statistical values of the mRNA levels. There was not significant change of Smad7 mRNA levels in the different group.

The expression of FN mRNA and Smurf2 mRNA increased in each experimental group compared with the NC group, especially in the diabetic nephropathy group (*P* < 0.01). There was however a decrease in FN and Smurf2 mRNA expression in the MH and ML groups compared with the diabetic nephropathy group (*P* < 0.05), and this decrease was more pronounced in the MH group compared with the ML group (*P* < 0.05) (Figure 5). The mRNA expression of Smad7 do not have any significant change in each group (*P* > 0.05) (Figure 6).

## 4. Discussion 

Currently, diabetic nephropathy has become the primary cause of end stage renal disease (ESRD) [17–19]. Previously, some studies found that a lot of cell signaling pathway could regulate the diabetic nephropathy fibrosis, such as NF-*κ*B, MAPK, TGF-*β* and so on [7, 20, 21].The TGF-*β* signaling pathway has been recognized as an important one in diabetic nephropathy fibrosis, its main biological function being to promote renal cell hypertrophy and regulate ECM metabolism. TGF-*β* signaling may inhibit cell proliferation by controlling cell transformation from Gl phase to S phase, inducing cell hypertrophy, increasing matrix synthesis, and decreasing matrix degradation, so as to promote the accumulation of ECM [11].

### 4.1. The Regulatory Role of the UPP in the TGF-*β* Signaling Pathway in Early Diabetic Nephropathy and the Relationship between the UPP and DN

The ubiquitin proteasome pathway (UPP) is an important nonlysosomal protein degradation pathway, which is widespread in eukaryotic cells. It is able to efficiently degrade intracellular proteins with high selectivity, which affects cell cycle regulation, apoptosis, antigen presentation, inflammatory reactions, and gene transcription [22, 23]. In particular, the UPP can upregulate or downregulate signaling pathways by degrading the intracellular inhibiting or activating factor of each pathway [24].

Smad7 is the key negative regulatory protein of the TGF-*β* signaling pathway, is located downstream of the TGF-*β* signaling pathway and is regulated by UPP [25, 26]. Smad7 can bind to the Smad ubiquitin regulatory factor 2 (Smurf2) for transfer to the cell membrane, followed by degradation of Smad2 and 3 and TGF-*β* receptor complexes, and inhibition of the activation of the TGF-*β* signaling pathway. Smurf2 is a ubiquitin ligase, which belongs to the E3 ligase family. It can specifically degrade Smad7 and weaken the inhibitory effect of Smad7 on the TGF-*β* receptor.

TGF-*β* activation plays an important role in the development of diabetic nephropathy, which may involve the expression of fibronectin (FN). A recent study reported that UPP was the main method for specific intracellular protein degradation. Furthermore, ubiquitin levels were found to be increased in type 2 diabetic neuropathy compared with the control group [27], suggesting that the development of diabetic nephropathy may be due to the degradation of neuroproteins. Kaniuk et al. [28] showed that high quantities of ubiquitin were found surrounding the pancreatic *β* cells in diabetic rats. In recent years, studies have determined that Smurf2 expression is increased in kidney disease models, for example, Smurf2 and TGF-*β* protein expression is increased after 7 days in the unilateral ureteral obstruction kidney, but expression is then decreased by the UPP inhibitor, MG132; at the same time, Smad7 expression is increased and fibrous degeneration is improved [29]. It has been reported that the UPP can reduce Smad7 expression in the kidney [6, 30]. These reports indicate that the UPP is involved in renal scarring.

However, whether ubiquitin degradation of Smad7, which regulates the TGF-*β* signaling pathway, is involved in the development of diabetic nephropathy by the UPP is unknown. In this study, we discovered that Smurf2, a member of the UPP family, increased in the diabetic nephropathy group, concomitantly with increased expression of TGF-*β* and FN, followed by decreased expression of Smad7. However, these effects were reduced by the UPP inhibitor, MG132. Results demonstrated that regulation of the TGF-*β* signaling pathway by Smad7 was involved in the development of DN. The UPP participated in the activation of the TGF-*β* pathway and induced the progress of diabetic nephropathy by ubiquitin degradation of Smad7.

### 4.2. The Therapeutic Effect of the Ubiquitin-Proteasome Inhibitor MG132 in Early Diabetic Nephropathy

Currently, the incidence of diabetic nephropathy is increasing annually, along with the increased incidence of diabetes. However, the mechanisms by which diabetic nephropathy develops are not fully elucidated, and the onset of diabetic nephropathy cannot be prevented despite strict control of blood glucose, blood pressure, and blood lipids due to monitoring dietary intake. Therefore, it is necessary to explore molecular mechanisms involved in DIABETIC NEPHROPATHY development in order to find a new therapeutic target.

A key catalytic enzyme, involved in ubiquitin degradation of target proteins in the UPP, is the 26s proteasome, which is specifically inhibited by the UPP inhibitor MG132 [11, 31–33]. Tashiro et al. discovered that this UPP inhibitor can alleviate renal interstitial fibrosis in unilateral ureteral obstruction nephrosis rats [31]. Recent studies have shown that MG132 can protect the kidney against diabetes-induced oxidative damage, inflammation, and fibrosis [13–15, 34], but the exact pathogenesis has not yet been completely clarified. Our previous study has found that MG132 could depress the activation of NF-*κ*B inflammatory signaling through inhibiting the I*κ*B*α* sumoylation and ubiquitination, and could inhibit the histone ubiquitination and induce apoptosis in rat glomerular mesangial cells induced by high glucose [13, 20] Renal fibrosis in diabetic nephropathy was induced by activation of the TGF-*β* signaling pathway, which mediates cell proliferation and differentiation, but whether the proteasome inhibitor could treat diabetic nephropathy by blocking ubiquitin degradation of Smad7 was not reported.

In our study, we found that administration of MG132 in diabetic nephropathy rats led to a decrease in body weight and Smad7 protein expression, while we did not observed the significant changes in the mRNA expression of Smad7. In addition, kidney weight, kidney weight index, UAER, Up/Ucr, TGF-*β* protein and FN mRNA levels decreased upon inhibition with MG132. The pathological changes upon observation with light and electron microscopes also reduced significantly in the MH group. Furthermore, MG132 can regulate the expression of Smurf2 mRNA in a concentration-dependent manner. This suggests that MG132, as a UPP inhibitor, can protect rat podocytes from damage, improve endothelial cell edema, maintain basement membrane permeability and reduce urinary protein. It can also inhibit Smurf2 expression, reduce ubiquitin degradation of Smad7, enhance the Smad7-induced inhibition of the TGF-*β* signaling pathway, and partially block TGF-*β* protein expression and FN mRNA to delay renal fibrosis. Moreover, we did not find it has obvious side effects on rats in our study, for example, the dysfunction of liver and kidney. Therefore, a novel mechanism in diabetic nephropathy may be the activation of the UPP, which increases ubiquitin degradation of Smad7, which is a inhibiting factor in the TGF-*β*/SMAD signaling pathway. MG132 can improve the early stages of diabetic nephropathy in rats by reducing diabetic renal pathological changes, improving nephropathy urine protein, partly lowering fibronectin expression and reducing renal fibrosis.

## 5. Conclusion

We have demonstrated that activation of the TGF-*β* signaling pathway is related to an increased ubiquitin degradation of the Smad7 protein by the UPP, in early DN. We have also shown that MG132 has a therapeutic effect on early diabetic nephropathy by blocking ubiquitin degradation of Smad7 and thus inhibiting activation of the TGF-*β* pathway.

## Figures and Tables

**Table 1:** Results of Body Weight, Kidney Weight, and Kidney Weight Index. Body weight was lower in the experimental groups compared with the NC group, whereas it was significantly recovered in the MH and ML groups compared with the DN group. Conversely, kidney weight and kidney weight index were greater in the experimental groups, but decreased upon MG132 treatment. Changes in the MH group were more significant than the ML group.

Group	Amount	BW (g)	KW (g)	IKW (10^−3^)
NC group	10	428.60 ± 20.74	3.04 ± 0.26	6.77 ± 0.90
DN group	10	214.40 ± 7.89^▲^	5.06 ± 2.13^▲^	45.32 ± 1.83^▲^
MH group	10	322.60 ± 25.89^▲■∗^	3.58 ± 2.31^▲●^	22.55 ± 2.7^▲●∗^
ML group	10	271.40 ± 28.17^▲■^	3.77 ± 1.43^▲●^	27.33 ± 2.97^▲●^

^▲^ *P* < 0.05 versus NC group, ^■^ *P* < 0.01 versus DN group, ^●^ *P* < 0.05 versus DN group,*P < 0.01 versus ML group.

**Table 2:** Results of UV, UAER, and Up/Ucr ($\overset{-}{x}\pm s$). The values of UV, UAER, and Up/Ucr were greater in the experimental groups compared with the NC group, and were the highest in the DN group. However, these values decreased in the MH and ML groups compared with the DN group, with a more pronounced decrease in the MH group compared with the ML group.

Group	Amount	UV (mL)	UAER (mg/24 h)	UP/UCR (g/gcr)
NC group	10	38.40 ± 3.78	3.21 ± 0.97	0.13 ± 0.03
DN group	10	169.75 ± 10.724^▲^	64.23 ± 11.45^▲^	0.54 ± 0.11^▲^
MH group	10	123.25 ± 24.6^▲●∗^	35.75 ± 10.06^▲●∗^	0.29 ± 0.04^▲●∗^
ML group	10	139.80 ± 14.87^▲●^	42.88 ± 4.43^▲●^	0.36 ± 0.06^▲●^

^▲^ *P* < 0.05 versus NC group, ^●^ *P* < 0.05 versus DN group, *P < 0.05 versus ML group.

**Table 3:** Results of Bcr, ALT, and AST. The values of Bcr, ALT, and AST do not change in each group (*P* > 0.05).

Group	Amount	Bcr (*μ*mmol/L)	ALT (U/L)	AST (U/L)
NC group	10	53.70 ± 4.01	18.3 ± 1.47	14.6 ± 1.04
DN group	10	57.5 ± 2.3	16.0 ± 1.72	16.1 ± 1.39
MH group	10	56.4 ± 5.53	20.1 ± 2.99	17.5 ± 1.82
ML group	10	54.9 ± 3.12	19.2 ± 1.86	16.9 ± 1.67
